# Spontaneous rupture of continent cutaneous urinary diversion after 25 years

**DOI:** 10.15171/jrip.2017.15

**Published:** 2016-11-19

**Authors:** Seyed Yousef Hosseini, Mehdi Dehghani, Amin Afsharimoghaddam, Zahra Sepehri, Mahdi Afshari

**Affiliations:** ^1^Urology and Nephrology Research Center and Department of Urology, Shahid Beheshti University of Medical Science, Tehran, Iran; ^2^Department of Urology, Zabol University of Medical Science, Zabol, Iran; ^3^Department of Internal Medicine, Zabol University of Medical Science, Zabol, Iran; ^4^Department of Epidemiology, Zabol University of Medical Science, Zabol, Iran

**Keywords:** Spontaneous rupture, Urinary diversion, Cutaneous pouch, Radical cystoprostatectomy

## Abstract

Spontaneous rupture of a continent cutaneous urinary diversion is uncommon and diagnosis of this situation requires a high degree of suspicion. In this paper we present a 66-year-old man with continent cutaneous pouch after radical cystoprostatectomy that presented with spontaneous perforation 25 years after surgery. Spontaneous pouch perforation in our case after 25 years emphasizes the need for long follow-up in patients with continent diversion.

**Implication for health policy/practice/research/medical education:**

Spontaneous rupture of a continent cutaneous urinary diversion is uncommon and diagnosis of this situation requires a high degree of suspicion and emphasizes the need for long follow-up in patients with continent diversion.

## Introduction

The 7th most common malignancy in males and 17th in females is bladder cancer (1,2). The best treatment in cases with localized muscle invasive tumor as well as cases with high grade non-muscle invasive bladder cancer is radical cystectomy (3-6).

While orthotopic bladder replacement is accepted as a diversion method, but continent cutaneous pouch for reconstruction of the lower urinary tract is a good option in suitable cases after radical cystectomy (7) and the use of detubularized ileum has wide acceptance (8).

Some complications which are specifically associated to catheterizable cutaneous pouch consist of catheterizing difficulties, stenosis of stoma, stones, stricture of ureterointestinal anastomosis, and ultimately pouch rupture. Majority of complications take place in the first three years after surgery however, rarely some events may occur many years after the operation (9,10).

Spontaneous perforation of continent cutaneous urinary diversion is an absolute rare event; yet it is a well-documented complication after reconstruction of lower urinary tract (11).

## Case Presentation

A 66-year-old man presented to the emergency ward complaining of fatigue, malaise and abdominal pain without signs of generalized peritonitis from two days ago. The patient was ill and conscious – but not toxic – without any fever or changes in blood pressure and heart rate. The patient experienced unwillingness drinking and eating and simultaneously decrease in urine output. Laboratory data showed increasing in blood urea nitrogen (BUN) and creatinine (Cr) with mild metabolic acidosis. Serum Cr: 1.9 mg/dL, BUN: 71 mg/dL, ph: 7.24, PHCO3: 11 mm Hg and Pco2 was 33 mm Hg. Ultrasonography of abdomen and pelvis revealed no hydronephrosis, however some intraabdominal free fluid was detected.

He had a continent cutaneous urinary diversion with detubularized ileum due to radical cystoprostatectomy 25 years ago after diagnosis of muscle invasive bladder cancer.

After the surgery, clean intermittent catheterization (CIC) has been conducted every 6 hours to make empty his neobladder. During the first year post-surgery, he has under follow up surveillance and there were no metastasis and tumor recurrence during this period, however he abandoned follow up and had experienced no problem for 25 years until 2 days ago.

**Figure 1:**
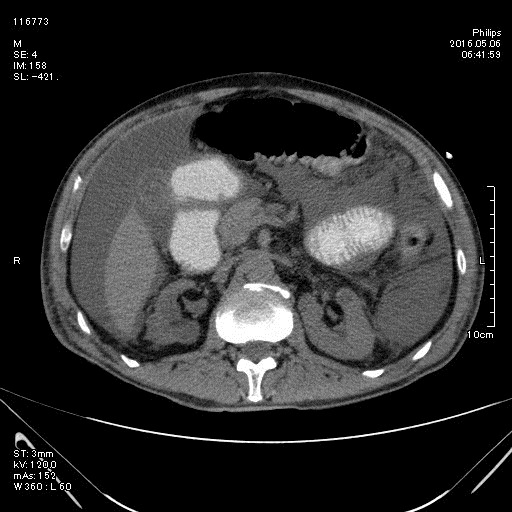
Intra-abdominal free fluid in the oral contrast-computed tomography without bowel obstruction.

**Figure 2:**
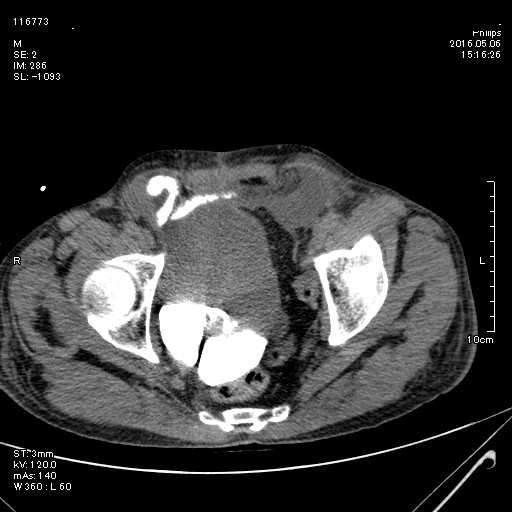
Eextravasation of contrast media in the CTpouchography

At entrance immediately hydration and antibiotic therapy started and a 16 Fr Foley catheter gently inserted in the pouch through the cutaneous stoma and ultimately only 500 mL of urine was drained. Examination revealed periumbilical pain and tenderness without rebound tenderness. Patient mentioned no defecation or gas passage during last 2 days. A computed tomography (CT) with oral contrast revealed no bowel obstruction, while it also indicated intraabdominal free fluid as like ultra-sonographic findings (Figure 1). The CT-pouchography was conducted and confirmed extravasation of contrast media from the pouch (Figure 2).

While on conservative managements, his abdominal distention and decreasing in urinary output was progressed, we decided to explore the abdomen. At laparotomy a 2 cm defect in left anterior side of the pouch was detected and repaired.

His postoperative course was passed without any complication and he discharged one week after surgery while, Foley catheter remained inside the pouch via the cutaneous stoma. After three weeks Foley catheter was removed and he continued CIC.

## Discussion

Complication rate is high in urinary diversion even after 5 years; Scott et al concluded that the patients should be informed of complications and they should be under followed up as tightly as for cancer follow up (12). Accordingly Richard et al, concluded that, radical cystectomy and subsequent urinary diversion is the most difficult urologic surgery, and the complications may occur even after 20 years (13).

Spontaneous rupture of continent urinary diversion is extremely rare late complication though in some studies this complication is not even reported at all (14). In fact, the exact incidence of spontaneous perforation of continent cutaneous urinary diversion is not really understood (15-18).

The rate of complications should be chronically evaluated while the proportion of patients decrease during the following years, hence it might be underestimated years or decades after surgery (13).

Mansson et al detected a higher rate of neobladder perforation in continent cutaneous urinary diversion than orthotopic pouches, while there is no pop-off mechanism in the former. The incidence of perforation in this report is 1.5% and there is no difference between colonic or ileal pouches in perforation rate. Although there was a perforation in appendiceal outlet in one case (16).

Most complications of radical cystectomy and urinary diversion is related to the urinary diversion section. Although the literature reports all complications are the same for the two procedures (15).

Some crucial contributing factors for rupture are possible weakening and ischemic changes of pouch wall due to acute or chronic over-distention of continent diversion (17).

Patients may present with some symptoms such as localized pain around stoma or mild to severe generalized abdominal pain or signs of frank peritonitis, sepsis, ileus and fever. Admittedly diagnosis needs a high degree of suspicion. Computed tomography with pouchography is possibly a better option for imaging.

Furthermore, several reports of conservative management with catheter drainage, and wide spectrum antibiotic therapy in literature were reported (18), however in the presence of generalized peritonitis or progressing and worsening patient’s condition (abdominal distention, fever, oliguria, etc.) laparotomy is mandatory.

## Conclusion

Spontaneous pouch perforation in our case after 25 years emphasizes the need for long follow-up in patients with continent diversion. Sudden onset of abdominal pain in a patient with continent pouch (orthotopic or cutaneous) should be considered rupture until proven otherwise. To our knowledge, this is the latest spontaneous pouch perforation that reported till now.

## Acknowledgments

Authors would like to thank Dr. Zohre Kiani for his invaluable comments on the manuscript.

## Authors’ contribution

SYH; conceived the study. MD; participated in design and coordination of the study. AFM; collected data and wrote first draft. ZS; helped in data collection and drafting the manuscript from beginning to the last point. MA; read and approved the final version of manuscript. All authors also read and approved the final manuscript.

## Conflicts of interest

The authors declare no conflict of interest.

## Ethical considerations

Ethical issues (including plagiarism, data fabrication, double publication) have been completely observed by the authors. Written consent was obtained from the patient for publication of the study.

## Funding/Support

None.
